# Expression Study and Clinical Correlations of *MYC* and *CCAT2* in Breast Cancer Patients

**DOI:** 10.18869/acadpub.ibj.21.5.303

**Published:** 2017-09

**Authors:** Shaghayegh Sarrafzadeh, Lobat Geranpayeh, Behnoosh Tasharrofi, Mohammad Soudyab, Elahe Nikpayam, Mostafa Iranpour, Reza Mirfakhraie, Jalal Gharesouran, Somayyeh Ghafouri-Fard, Soudeh Ghafouri-Fard

**Affiliations:** 1 Department of Medical Genetics, Shahid Beheshti University of Medical Sciences, Tehran, Iran; 2 Department of Surgery, Sina Hospital, Tehran University of Medical Sciences, Tehran, Iran; 3 Department of Medical Genetics, Faculty of Medicine, Tabriz University of Medical Sciences, Tabriz, Iran; 4 Department of Community and Preventive Medicine, School of Medicine, Tehran University of Medical Sciences, Tehran, Iran

**Keywords:** Long non-coding RNA, Breast cancer, c-MYC

## Abstract

**Background:**

Colon cancer-associated transcript 2 (*CCAT2*) is a newly recognized lncRNA transcribed from the 8q24 genomic region. It functions as an oncogene in various types of cancers including breast cancer, in which it affects Wnt/β-catenin pathway. Previous studies have shown a putative interaction between this lncRNA and *MYC* proto-oncogene.

**Methods:**

In the current study, we evaluated the expression of *CCAT2* in breast cancer tissues with regards to the expression of its target *MYC*. In addition, we assessed the relationship between *CCAT2* and *MYC* expression levels in tumor tissues and the clinical prognostic characteristics of breast cancer patients.

**Results:**

*MYC* expression levels were significantly up-regulated in tumor tissues compared with adjacent non-cancerous tissues (ANCTs), while such analysis showed no statistically significant difference between these two tissue types in *CCAT2* expression. Starkly increased *CCAT2* gene expression levels were found in 12/48 (25%) of cancer tissue samples compared with their corresponding ANCTs. Furthermore, significant inverse correlations were found between *CCAT2* expression and stage, as well as lymph node involvement. Besides, a significant inverse correlation was found between the relative *MYC* expression in tumor tissues compared with their corresponding ANCTs and disease stage.

**Conclusions:**

These results highlight the significance of *MYC* and *CCAT2* expressions in the early stages of breast cancer development and suggest a potentially significant role for *CCAT2* in a subset of breast cancer patients, which could be applied as a potential therapeutic target in these patients.

## INTRODUCTION

Accumulating evidence suggests that a group of non-coding RNAs ranging in size from 200 to 1000 nucleotides participates in tumorigenesis processes[1]. Such long non-coding RNAs (lncRNAs) have been shown to control critical pathways for tumor initiation and progression. The lncRNAs have a tissue-specific function and tumor stage-specific expression, which potentiate them as important biomarkers and therapeutic targets[2]. Consequently, researchers have focused on the identification of their tumor-specific signature in several cancer types[3] including breast cancer[4,5].

Breast tumors are among cancers with frequent gene copy number aberrations. Among cytogenetic abnormalities detected in breast tumors, amplification of oncogene(s) located on 8q24 has been suggested to participate in the development and/or progression of a great number of primary breast cancers, principally invasive ones[6,7].

Colon cancer-associated transcript 2 (*CCAT2*) is a recently identified lncRNA transcribed from the 8q24 genomic region[8]. This lncRNA has been regarded as an oncogene in several kinds of cancers including breast and colon cancer, in which it exerts its function via affecting Wnt/β-catenin pathway[8,9]. The *CCAT2* genomic locus contains certain polymorphisms that have been demonstrated to be associated with susceptibility to various cancers[8], as well as with the risk of metastasis in inflammatory breast cancer[10]. In addition, among these polymorphisms, G-allele genotype of rs13281615 has been regarded as a risk factor for developing breast cancer, while the AA genotype has been shown to protect against its occurrence[11].

Previously, it has been noted that each risk locus in 8q24 region has epigenetic marks consistent with enhancer elements and forms a chromatin loop with the *MYC* proto-oncogene, which localizes numerous 100 kb telomerics to this region. Consequently, the 8q24 risk loci has been suggested as tissue-specific enhancers of *MYC*[12]. Previous independent reports have shown the overexpression of *CCAT2* in breast cancer tissues[2], as well as overexpression of *MYC* in certain types of breast cancer[13]. Although the role of *CCAT2* in up-regulation of *MYC* expression has been demonstrated in colon cancer *in vitro*[8], there is no sufficient data regarding the possible interactions of these two genes in the tumorigenesis process in breast cancer samples. As a result, in the current study, we assessed the expression of *CCAT2* in breast cancer tissues with regard to the expression of its target *MYC*. Subsequently, we evaluated the relationships between *CCAT2* and *MYC* expression levels in tumor tissues and the clinical prognostic characteristics of breast cancer patients.

## MATERIALS AND METHODS

### Patients’ samples

The Ethical Committee of Shahid Beheshti University of Medical Sciences (Tehran, Iran) approved this study. All the samples were acquired with the patients’ informed consent. Paired breast cancer tissue and adjacent normal breast tissue were obtained from 48 patients who had undergone surgical breast cancer resection at the Departments of Surgery in the Tehran University of Medical Sciences affiliated hospitals between 2014 and 2016. All the participants were female with the average age of 51.35±14.721. Early onset cases were excluded from the study. Breast cancer was diagnosed based on American Society of Clinical Oncology breast cancer guidelines. The non-tumorous tissue samples were at least two cm from the edge of the tumor, comprised no apparent tumor cells, and were assessed by the pathologists. The tissue samples were frozen in liquid nitrogen immediately after surgical removal and stored at -80°C.

### RNA extraction and quantitative RT-PCR

Total RNA was extracted from tissue samples using the AccuZol™ total RNA extraction solution (Bioneer, Korea) according to the manufacturer’s instructions. RNA purity and concentration were quantified by Thermo Scientific NanoDrop™ 1000 Spectrophotometer (Waltham, MA, USA). RNA (1 µg) was used in cDNA synthesis by using PrimeScript RT reagent kit (Takara Bio, Ohtsu, Japan). Quantitative RT-PCR reaction was performed on a Rotor-Gene 6000 Corbett detection system using SYBR Premix Ex Taq (Takara Bio, Ohtsu, Japan). Thermal cycling conditions were an initial activation step at 95º C for 5 minutes, followed by 40 cycles of 95ºC for 15 seconds, specific annealing temperature for 20 seconds and an extension step at 72ºC for 20 seconds, followed by melting curve acquisition. Specific annealing temperatures for *MYC*, *CCAT2*, and *B2M* were 56.5ºC, 58.5ºC, and 60ºC, respectively. We also included no template control consisting of H_2_O in each run. *B2M* gene was used for normalization of RT-PCR data as indicated in a previous study[4]. The sequences of forward and reverse primers sequences are listed in Table 1.

**Table 1:** The nucleotide sequences for forward and reverse primers

Gene	Forward primer	Reverse primer
*CCAT2*	5’-AAGAGGGAGGTATCAACAGAGAC-3’	5’-TTTGGACGACGCCTTCATTTC-3’
*MYC*	5’-CACATCAGCACAACTACG-3’	5’-GTTCGCCTCTTGACATTC-3’
*B2M*	5’-AGATGAGTATGCCTGCCGTG-3’	5’-GCGGCATCTTCAAACCTCCA-3’

### Estrogen receptor (ER)/progesterone receptor (PR)

The ER/PR status of each patient was confirmed by immunohistochemical (IHC) staining and obtained from patients’ medical records. Staining of >20% of tumor cell nuclei was regarded as positive and that of 5% to 19% as borderline. When <5% of tumor cell nuclei was stained, a negative result was reported. Both borderline and obviously positive results were considered as positive.

### Human epidermal growth factor receptor 2 (HER2/neu)

HER2/neu results were obtained from the medical record and accomplished by IHC assay. For the present study, a test result of 0-2+ was considered as negative and 3+ as positive.

### Ki-67

Ki-67 status was evaluated using IHC assay. Ki-67 values were reported as the percentage of positively marking malignant cells among the total number of malignant cells assessed using the anti-human Ki-67 monoclonal antibody MIB1 or alternatively as positive vs. negative.

### Statistical analysis

Fold changes in gene expression were quantified by LinRegPCR (version 2) and Relative Expression Software Tool-RG©-version 3 (QIAGEN, Korea) by means of the amplification efficiencies and cycle thresholds from comparative quantification analysis. By the means of LinRegPCR, the baseline fluorescence was determined and subtracted and PCR efficiencies were then computed for each sample. Afterwards, Cq value and the starting concentration per sample (reported in an arbitrary unit) were measured. The calculated Cq and efficiency values were used for quantification analysis. The quantities of mRNAs in the tissues were standardized to the *B2M* mRNA and compared between tumor and non-cancerous tissues. The pairwise fixed reallocation randomization test with 2000 iterations in the REST 2009 software was used to express the significances. The level of statistical significance was set at *P*<0.05. The associations of demographic and clinical data with gene expression levels were assessed using SPSS v.18.0.1 (SPSS Inc., Chicago, IL, USA). The data were presented as the mean±SD. The McNemar’s test was used to compare paired tumor and adjacent non-cancerous tissues (ANCTs). Chi-square and independent *t*-tests were applied to assess the significance of *CCAT2* expression as correlated with clinicopathologic features in breast cancer. Significance was delineated as *P*<0.05.

## RESULTS

### General statistical information

Data were analyzed according to the evidence obtained from questionnaires, interviews, as well as clinical and laboratory tests. Table 2 demonstrates the demographic and clinical data of patients, including age, histological features, tumor size, tumor grade, and stage.

**Table 2:** Demographic and clinical data of patients

Characteristics	Values
Age (mean±SD)	51.35±14.721 (35-84)
Menarche age (mean±SD)	13.41±1.117 (12-16)
Menopause age (mean±SD)	49.84±4.413 (38-59)
First pregnancy age (mean±SD)	21.41±5.056 (14-34)
Breast feeding duration (months) (mean±SD)	50.20±52.206 (0-240)
Positive family history for cancer (%)	25.6
**Body mass index (%)**	
<18.5	7.1
18.5-24.9	57.1
>25	35.7
**History of oral contraceptive use (%)**	
Yes	67.4
No	32.6
**History of hormone replacement therapy after menopause (%)**	
Yes	18.6
No	81.4
**Cancer stage (%)**	
I	7
II	58.1
III	30.2
IV	4.7
**Overall grade (%)**	
I	11.9
II	54.8
III	33.3
**Nuclear grade (%)**	
I	7.1
II	54.8
III	38.1
**Tubule formation (%)**	
I	7.1
II	38.1
III	54.8
**Mitotic rate (%)**	
I	33.3
II	50
III	16.7
**Tumor size (%)**	
<2 cm	16.3
≥2 cm, <5 cm	76.7
≥5 cm	7
**Estrogen receptor (%)**	
Positive	70.7
Negative	29.3
**Progesterone receptor (%)**	
Positive	63.4
Negative	36.6
**Her2/neu expression (%)**	
0	26.8
1	22
2	26.8
3	24.4
**Ki67 expression (%)**	
Positive	94.7
Negative	5.3

### Expression of *CCAT2* and *MYC* in patients’ samples

**Fig. 1:**
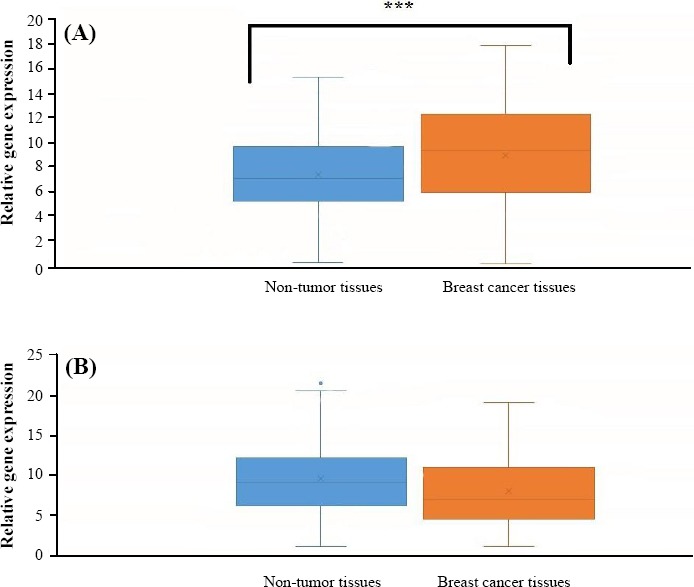
Comparison of genes expression levels between total tumor and adjacent non-cancer tissues. (A) *MYC* relative expression levels; (B) *CCAT2* relative expression levels. ^*^P>0.05; ^***^P<0.05

**Fig. 2:**
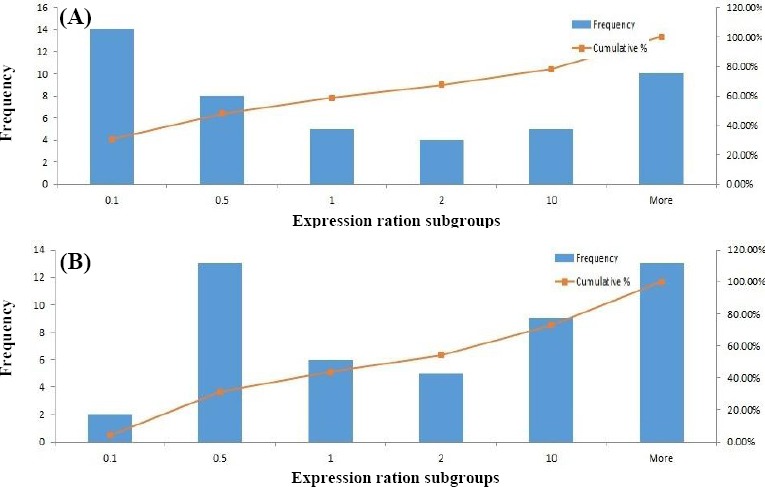
Frequency and cumulative percentage of samples in each subgroup based on relative expression of *CCAT2* (A) and *MYC* (B) in tumor tissues compared with adjacent non-cancer tissues.

Comparison of *MYC* expression levels between total tumor and ANCT tissues showed a significant up-regulation in tumor tissues (*P*=0.02) (Fig. 1A). However, such analysis showed no statistically significant difference between these two tissue types in *CCAT2* expression (*P*>0.05) (Fig. 1B). Starkly increased *CCAT2* gene expression levels were observed in 12/48 (25%) of cancer tissue samples, as compared with their corresponding ANCTs. Figure 2 shows the frequency and cumulative percentage of samples in each subgroup based on relative expression of *CCAT2* and *MYC* in tumor tissues compared with adjacent non-cancer tissues.

### Correlations between *CCAT2* expression and clinical characteristics

To further discover the role of *CCAT2* in breast cancer, we subsequently assessed the associations between its transcript levels and several clinicopathological characteristics. A significant inverse relationship was found between *CCAT2* expression and stage, as well as lymph node involvement. The relationship between *CCAT2* expression and clinicopathological variables is shown in Table 3. The 48 patients were then divided into two groups by the median value of relative *CCAT2* transcript levels in tumor tissues compared with the corresponding ANCT: high (n=12) and low (n=36) expression groups. Statistical analyses between these two groups showed a significant inverse association between lymph node involvement and *CCAT2* transcript levels (*P*=0.02). No significant correlation was found between transcript levels and other clinicopathological variables. Further analyses were performed in patients with low *CCAT2* transcript levels in tumor tissues, as compared with the corresponding ANCT, by dividing into two subgroups based on the relative transcript levels (>0.5 and <0.5). No other significant relationship was found between *CCAT2* transcript levels and clinicopathological variables.

**Table 3:** *CCAT2* expression and its associations with patients’ clinical and demographic data

Characteristics	Down-regulation	Up-regulation	N	*P*
**Age**				0.205
<50	12	12	24	
>=50	14	7	21	
**Stage**				0.001
Early stages (0, I, II)	12	18	30	
Advance stages (III, IV)	14	1	15	
**Histological Grade**				0.74
I	3	2	5	
II	14	10	24	
III	8	7	15	
**Tumor Size**				0.705
<2	5	2	7	
≥2 cm, <5 cm	19	14	33	
≥5 cm	2	1	3	
**Node Status**				0.015
Negative	11	15	26	
Positive	15	4	19	
**ER Status**				0.525
Negative	6	5	11	
Positive	19	13	32	
**PR Status**				0.409
Negative	9	5	14	
Positive	16	13	31	
**HER2/neu Status**				0.475
Negative	18	14	32	
Positive	7	4	11	
**Ki67 Status**				0.348
Negative	1	23	24	
Positive	2	14	16	

N, number; ER, estrogen receptor; PR, progesterone receptor; HER2/neu, human epidermal growth factor receptor 2

### Correlations between *MYC* expression and clinical characteristics

The relationship between *MYC* expression and clinicopathological variables is shown in Table 4. A significant inverse correlation was found between the relative *MYC* expression in tumor tissues in comparison with their corresponding ANCTs and disease stage. We further divided the 48 patients into two groups by the median value of relative *MYC* transcript levels in tumor tissues compared with the corresponding ANCT: high (n=14) and low (n=34) expression groups. Statistical analyses between these two groups showed a significant inverse association between *MYC* transcript levels and disease stage (*P*=0.030), as well as the tumor size (*P*=0.011). No significant association was found between transcript levels and other clinicopathological variables. Further analyses were performed in patients with low *MYC* transcript levels in tumor tissues compared with the corresponding ANCT by division into two subgroups based on the relative transcript level (>0.5 and <0.5). Such analyses showed a significant inverse correlation between histological grade and *MYC* relative expression level (*P*=0.007).

**Table 4:** *MYC* expression and its associations with patients’ clinical and demographic data

Characteristics	Down-regulation	Up-regulation	N	*P*
**Age**				0.424
<50	12	13	25	
>=50	9	13	22	
**Stage**				0.039
Early stages (0, I, II)	11	21	32	
Advance stages (III, IV)	10	5	15	
**Histological Grade**				0.621
I	2	4	6	
II	11	14	25	
III	7	8	15	
**Tumor Size**				0.152
<2	2	6	8	
≥2 cm, <5 cm	17	17	34	
≥5 cm	2	1	3	
**Node Status**				0.273
Negative	11	17	28	
Positive	10	9	19	
**ER Status**				0.547
Negative	5	15	20	
Positive	7	18	25	
**PR Status**				0.460
Negative	6	9	15	
Positive	14	16	30	
**HER2/neu Status**				0.167
Negative	17	17	34	
Positive	3	8	13	
**Ki67 Status**				0.391
Negative	2	1	3	
Positive	16	23	39	

N, number; ER, estrogen receptor; PR, progesterone receptor; HER2/neu, human epidermal growth factor receptor 2

### Relative expression of *CCAT2* and *MYC* in individual samples

In order to determine any correlation between the expressions of these two genes, the relative expression of these genes was compared in each set of samples. No significant association was observed between the levels of transcripts in tumor tissues (R^2^=0.065, *P*>0.05) or ANCTs (R^2^=0.17, *P*>0.05).

## DISCUSSION

Identification of tumor-specific signature of non-coding RNAs, which are correlated with cancer detection, patient prognosis, and response to therapy, is the aim of many recent studies. In the present investigation, we evaluated the expression of *CCAT2* in breast cancer tissues in comparison with ANCTs and showed a significant *CCAT2* overexpression in tumor samples in a subset of patients. However, in other patients, the level of *CCAT2* transcripts was lower in tumor tissues compared with the corresponding ANCTs. This reduction can be explained by the high level of heterogeneity among breast cancer patients, which has been also highlighted in the Redis *et al*.[2] study. They showed *CCAT2* overexpression in two out of three sets of patients and the correlation between transcript levels and clinical factors only for a subgroup of breast cancer patients. They also indicated *CCAT2* expression in epithelial cells of both tumor and unmatched non-tumor breast tissues, by means of *in situ* hybridization with a higher expression in the former. Redis et al.’s[2] study demonstrated that the higher levels of *MYC* expression in breast tumor samples were positively associated with *CCAT2*, and the *CCAT2* expression level was suggested as a predictor of metastasis and poor survival for a particular subgroup of breast cancer patients. In addition, in this subgroup of patients, a significant inverse correlation was detected between *CCAT2* levels and ER and PR levels unlike in our study that no association was found. Another study has shown *CCAT2* up-regulation in ovarian cancer samples compared with normal ovarian tissues and suggested a possible association with tumor progression and development[14].

In the present study, we demonstrated significant inverse relationship between *CCAT2* expression and disease stage, as well as lymph node status. Of note, the higher expression of *CCAT2* was associated with early stages and negative lymph node status. Considering the role of *CCAT2* as an oncogene, these data suggest that it might participate in the early stages of tumor development rather than late stages. The same association was found between *MYC* expression and disease stage, which is in accordance with the results of a previous study in breast cancer patients. Based on the mentioned study, MYC expression and *MYC* amplification were more commonly detected in early-onset compared to late-onset tumors[15]. The inverse correlations observed between transcript levels of both studied genes in the present study and disease stage are in accordance with the parallel expression of *CCAT2* and *MYC* expressions in breast tumors, which has been observed in a previous study[2]. The role of *MYC* in breast cancer has been highlighted by the participation of its target genes in cell growth, transformation, angiogenesis, and cell-cycle control, and also the fact that breast cancer 1 protein is involved in transcriptional regulation via interaction with *MYC*[16]. MYC amplification has been shown to be considerably associated with aggressive tumor phenotypes and poor patients’ survival. However, the relationship between amplification and overexpression is not obviously described[16]. In the present study, we assessed the relative expression level of *MYC* in tumor tissues regardless of its genomic amplification. Although a previous report has evaluated the associations between 8q24 region amplification and *CCAT2* expression in breast cancer patients[2], to our knowledge, there is no data regarding the association between *CCAT2* expression and precise *MYC* copy number, which should be evaluated in future studies.

*CCAT2* knock-down has resulted in the inhibition of cell proliferation and invasion *in vitro* and tumor formation *in vivo*[9]. In addition, its knock-down has impeded the Wnt/β-catenin signaling pathway transcriptional activity[9]. Considering the fundamental role of this pathway in the development of various cancers and its critical role in breast cancer[17], the expression analysis of *CCAT2* in breast cancer would pave the way for identification of the mechanism of aberrant signaling in breast cancer and designing more effective therapeutic strategies. The significant difference between *CCAT2* expression levels in the studied patients indicates that treatment strategies should be designed based on information obtained from each patient. This observation is in accordance with the differences found in therapeutic responses, even between patients suffered from a certain subtype of breast cancer[18]. Regarding the advent of signal transduction therapies in breast cancer[19], identification of biomarkers in each signaling pathway, which could predict the response to a specific treatment modality, is of practical significance. As different cancer therapies are effective in distinct subgroups of patients, it is necessary to find new predictive and prognostic markers to enhance the outcomes of treatments[20]. Taken together, these data suggest a potentially significant role for *CCAT2* in a subset of breast cancer patients, which could applied as a potential therapeutic target in these patients.

## References

[1] Soudyab M, Iranpour M, Ghafouri-Fard S (2016). The role of long non-coding rnas in breast cancer. Archives of Iranian medicine.

[2] Redis RS, Sieuwerts AM, Look MP, Tudoran O, Ivan C, Spizzo R, Zhang X, de Weerd V, Shimizu M, Ling H, Buiga R, Pop V, Irimie A, Fodde R, Bedrosian I, Martens JW, Foekens JA, Berindan-Neagoe I, Calin GA (2013). CCAT2, a novel long non-coding RNA in breast cancer:expression study and clinical correlations. Oncotarget.

[3] Nikpayam E, Tasharrofi B, Sarrafzadeh S, Ghafouri-Fard S (2017). The role of long non coding RNAs in ovarian cancer. Iranian biomedical journal.

[4] Iranpour M, Soudyab M, Geranpayeh L, Mirfakhraie R, Azargashb E, Movafagh A, Ghafouri-Fard S (2016). Expression analysis of four long noncoding RNAs in breast cancer. Tumour biology.

[5] Tasharrofi B, Soudyab M, Nikpayam E, Iranpour M, Mirfakhraie R, Sarrafzadeh S, Geranpayeh L, Azargashb E, Sayad A, Ghafouri-Fard S (2016). Comparative expression analysis of hypoxia-inducible factor-alpha and its natural occurring antisense in breast cancer tissues and adjacent noncancerous tissues. Cell biochemistry and function.

[6] Rennstam K, Ahlstedt-Soini M, Baldetorp B, Bendahl PO, Borg A, Karhu R, Tanner M, Tirkkonen M, Isola J (2003). Patterns of chromosomal imbalances defines subgroups of breast cancer with distinct clinical features and prognosis. A study of 305 tumors by comparative genomic hybridization. Cancer research.

[7] Yokota T, Yoshimoto M, Akiyama F, Sakamoto G, Kasumi F, Nakamura Y, Emi M (1999). Frequent multiplication of chromosomal region 8q24.1 associated with aggressive histologic types of breast cancers. Cancer letters.

[8] Ling H, Spizzo R, Atlasi Y, Nicoloso M, Shimizu M, Redis RS, Nishida N, Gafa R, Song J, Guo Z, Ivan C, Barbarotto E, De Vries I, Zhang X, Ferracin M, Churchman M, van Galen JF, Beverloo BH, Shariati M, Haderk F, Estecio MR, Garcia-Manero G, Patijn GA, Gotley DC, Bhardwaj V, Shureiqi I, Sen S, Multani AS, Welsh J, Yamamoto K, Taniguchi I, Song MA, Gallinger S, Casey G, Thibodeau SN, Le Marchand L, Tiirikainen M, Mani SA, Zhang W, Davuluri RV, Mimori K, Mori M, Sieuwerts AM, Martens JW, Tomlinson I, Negrini M, Berindan-Neagoe I, Foekens JA, Hamilton SR, Lanza G, Kopetz S, Fodde R, Calin GA (2013). CCAT2, a novel noncoding RNA mapping to 8q24, underlies metastatic progression and chromosomal instability in colon cancer. Genome research.

[9] Cai Y, He J, Zhang D (2015). Long noncoding RNA CCAT2 promotes breast tumor growth by regulating the Wnt signaling pathway. OncoTargets and therapy.

[10] Bertucci F, Lagarde A, Ferrari A, Finetti P, Charafe-Jauffret E, Van Laere S, Adelaide J, Viens P, Thomas G, Birnbaum D, Olschwang S (2012). 8q24 cancer risk allele associated with major metastatic risk in inflammatory breast cancer. PloS one.

[11] Gong WF, Zhong JH, Xiang BD, Ma L, You XM, Zhang QM, Li LQ (2013). Single Nucleotide Polymorphism 8q24 rs13281615 and Risk of Breast Cancer:Meta-Analysis of More than 100,000 Cases. Plos One.

[12] Ahmadiyeh N, Pomerantz MM, Grisanzio C, Herman P, Jia L, Almendro V, He HH, Brown M, Liu XS, Davis M, Caswell JL, Beckwith CA, Hills A, Macconaill L, Coetzee GA, Regan MM, Freedman ML (2010). 8q24 prostate, breast, and colon cancer risk loci show tissue-specific long-range interaction with MYC. Proceedings of the National academy of sciences.

[13] Xu J, Chen Y, Olopade OI (2010). MYC and breast cancer. Genes and cancer.

[14] Huang S, Qing C, Huang Z, Zhu Y (2016). The long non-coding RNA CCAT2 is up-regulated in ovarian cancer and associated with poor prognosis. Diagnostic pathology.

[15] Pereira CB, Leal MF, de Souza CR, Montenegro RC, Rey JA, Carvalho AA, Assumpcao PP, Khayat AS, Pinto GR, Demachki S, de Arruda Cardoso Smith M, Burbano RR (2013). Prognostic and predictive significance of MYC and KRAS alterations in breast cancer from women treated with neoadjuvant chemotherapy. PLoS One.

[16] Chen Y, Olopade OI (2008). MYC in breast tumor progression. Expert review of anticancer therapy.

[17] Taherian-Esfahani Z, Abedin-Do A, Nouri Z, Mirfakhraie R, Ghafouri-Fard S, Motevaseli E (2016). *Lactobacilli* differentially modulate mTOR and Wnt/β-catenin pathways in different cancer cell lines. Iranian journal of cancer prevention.

[18] Yersal O, Barutca S (2014). Biological subtypes of breast cancer:Prognostic and therapeutic implications. World journal of clinical oncology.

[19] Jang GB, Kim JY, Cho SD, Park KS, Jung JY, Lee HY, Hong IS, Nam JS (2015). Blockade of Wnt/beta-catenin signaling suppresses breast cancer metastasis by inhibiting CSC-like phenotype. Scientific reports.

[20] Weber WA (2009). Assessing tumor response to therapy. Journal of nuclear medicine.

